# Persistence and variability of Stenotrophomonas maltophilia in cystic fibrosis patients, Madrid, 1991-1998.

**DOI:** 10.3201/eid0701.010116

**Published:** 2001

**Authors:** S Valdezate, A Vindel, L Maiz, F Baquero, H Escobar, R Cantón

**Affiliations:** Hospital Ramón y Cajal, Madrid, Spain.

## Abstract

During 1991 to 1998 at least one Stenotrophomonas maltophilia pulmonary infection was observed in 25 (24%) of 104 cystic fibrosis patients at the same unit of our hospital in Spain. Ribotyping and pulse-field gel electrophoresis (PFGE) characterization of 76 S. maltophilia isolates from these patients indicated an overall clonal incidence of 47.1%, reflecting new strains in 44% of patients with repeated positive cultures for S. maltophilia. Six patients with repeated episodes were persistently colonized (> or = 6 months) with the same strain. S. maltophilia bacterial counts were higher (geometric mean, 2.9 x 10(8) cfu/mL) in patients with repeated episodes than in those with a single episode (8.4 x 10(4) cfu/mL, p < 0.01). Single episodes of S. maltophilia occurred in patients < 10 years of age (43% [6/14]), whereas chronic colonization occurred more frequently in older patients (> 16 years of age).


### Persistence and Variability of *Stenotrophomonas maltophilia* in Cystic Fibrosis Patients, Madrid, 1991–1998

**Sylvia Valdezate,*,† Ana Vindel,† Luis Maiz,* Fernando Baquero,* Hector Escobar,* and Rafael Cantón***
*Hospital Ramón y Cajal, Madrid, Spain; †Centro Nacional de Microbiología, Instituto Carlos III, Madrid, Spain

During 1991 to 1998 at least one *Stenotrophomonas maltophilia* pulmonary infection was observed in 25 (24%) of 104 cystic fibrosis patients at the same unit of our hospital in Spain. Ribotyping and pulse-field gel electrophoresis (PFGE) characterization of 76 *S. maltophilia* isolates from these patients indicated an overall clonal incidence of 47.1%, reflecting new strains in 44% of patients with repeated positive cultures for *S. maltophilia*. Six patients with repeated episodes were persistently colonized (≥6 months) with the same strain. *S. maltophilia* bacterial counts were higher (geometric mean, 2.9 x$10^8$ cfu/mL) in patients with repeated episodes than in those with a single episode (8.4 x$10^4$ cfu/mL, p<0.01). Single episodes of *S. maltophilia* occurred in patients <10 years of age (43% [6/14]), whereas chronic colonization occurred more frequently in older patients (>16 years of age).

Pulmonary infection due to chronic microbial colonization is the major cause of illness and death in cystic fibrosis (CF) patients. Mucoid *Pseudomonas aeruginosa,* which is involved in pulmonary damage, is the most frequently recovered pathogen. In contrast, little information is available about the role of other nonfermentative gram-negative rods. An increasing incidence of *Stenotrophomonas maltophilia* isolates has been reported in some CF centers during the last decade (1-4). Although an association between *S. maltophilia* colonization and lung damage has been observed (2,3), the role of the organism is still undetermined (5,6). In non-CF patients (e.g., immunocompromised or intensive-care unit patients), exposure to wide-spectrum antimicrobial drugs, long-term antimicrobial therapy, previous pulmonary infections, and chronic respiratory disease contribute to *S. maltophilia* acquisition and increase the risk for respiratory infection with this organism (7,8). All these risk factors are present in the CF population.

Address for correspondence: Rafael Cantón, Servicio de Microbiología, Hospital Ramón y Cajal, Carretera de Colmenar, Km 9,100, 28034-Madrid, Spain; fax: 34-91-3368809; e-mail: rcanton@hrc.insalud.es.

We analyzed *S. maltophilia* from respiratory isolates of 25 CF patients of the same CF unit during an 8-year period to determine a) the overall and yearly incidence of *S. maltophilia* infection or colonization and incidence as determined by molecular typing, ribotyping, and pulsed-field gel electrophoresis (PFGE); b) the age distribution of acquisition of *S. maltophilia* pulmonary infection or colonization in patients with single or repeated episodes; c) the persistence and variability of *S. maltophilia* isolates in patients who had more than one episode and the degree of genomic similarity identified among clones; and d) the epidemiologic link between similar isolates from different patients. We also investigated pulmonary function and other clinical aspects of *S. maltophilia*-infected or colonized patients.

### Materials and Methods

From 1991 to 1998, 25 CF patients (12 female and 13 male) of 104 who were clinically and microbiologically followed at the Hospital Ramón y Cajal CF Unit had at least one positive respiratory culture for *S. maltophilia*. CF was diagnosed by a positive sweat chloride test

(>60 mEq/L) in association with typical pulmonary and gastrointestinal findings or a positive family history. The age range of patients was <1 to 32 years (median 14.5 years). Eleven patients were homozygotes and eight heterozygotes for ΔF508, the most common mutation in the cystic fibrosis transmembrane conductance regulator (CFTR) gene, and one patient was negative for ΔF508. Mutation in this gene could not be determined in five patients. The mean number of sputum samples examined was 6.7 specimens per patient per year. All 25 patients were followed for at least 1 year during the study period (range 1 to 8 years, mean 5.8 years). Culture results were used to establish age at acquisition of *S. maltophilia*. When available, retrospective cultures obtained before 1991 were also taken into account.

*S. maltophilia* colonization in CF patients was considered persistent if positive cultures were obtained for >6 months, regardless of bacterial counts. Overall incidence was defined as the number of patients infected or colonized with *S. maltophilia*, independent of the number of positive cultures during the study period. The denominator was the total number of patients seen at the CF unit (104 patients). Yearly incidence was defined as the number of patients with new episodes of *S. maltophilia* infection or colonization, with the denominator the number of patients seen per year in the CF unit. The overall incidence and yearly incidence were recalculated when molecular typing data were available. These values were defined as overall clonal incidence and yearly clonal incidence, respectively, which represent the incidence of *S. maltophilia* episodes caused by different clonal strains.

### Bacteriologic Study and *S. maltophilia* Isolates

Respiratory secretions, mostly expectorated sputum, were homogenized with N-acetylcysteine and processed by a modified quantitative technique (9). Columbia 5% blood, MacConkey, mannitol and salt, and a selective *Burkholderia cepacia* agar media were incubated in air for 24 hours at 37°C, followed by 24 hours at 25°C. In addition, bacitracin-chocolate agar was plated and incubated in 5% $CO_2$ for 48 hours and Sabouraud-chloramphenicol and Sabouraud-chloramphenicol-cyclohexamide agar media for 4 weeks at 30°C and 37°C. A culture for *S. maltophilia* was considered positive when any growth of this organism was observed, regardless of bacterial count. Biochemical identification of *S. maltophilia* isolates was performed both with the API 20NE gallery (BioMerieux, Marcy-l´Étoile, France) and the semiautomatic PASCO system (Difco, Detroit, MI). Bacterial counts and co-colonization with other respiratory pathogens were also considered in the analysis. The same microbiologic protocol was applied to all patients, regardless of clinical condition.

### Ribotyping

DNA from all *S. maltophilia* isolates was prepared by treatment with hexadecyltrimethylammonium bromide (10). Ribotyping was performed as described (11). *Bam*H1, *Bsu*15I, *Eco*RI, and *Hin*dIII restriction endonucleases (Roche Diagnostic, Mannheim, Germany) were also tested in a representative number of isolates. The best-defined restriction pattern with a higher number of bands was observed with *Bam*H1 and *Hin*dIII. Digoxigenin-labeled phage λ*Hin*dIII-digested DNA (Roche) was used as a molecular size marker. DNA fragments were separated by electrophoresis in 0.7% agarose gels and were blotted onto nylon membranes. Membranes were hybridized with a digoxigenin-labeled rRNA probe with 16S+23S rRNA sequences of *Escherichia coli* (Roche) at 68ºC for 18 hours (12). Differences in numbers and the position of bands were considered.

### Pulsed-Field Gel Electrophoresis

*S. maltophilia* DNA was prepared and contained in agarose plugs for digestion with 30 U of *Xba*I (Roche). Closely related isolates using *XbaI* were reanalyzed with 20 U of *Spe*I (Roche) as described (13). Digested samples were melted and loaded onto 1% agarose gels. PFGE was performed with the CHEF-DRII system (Bio-Rad, Hemel Hempstead, UK). Standard lambda ladders comprising 48.5-kbp concatemers were run as molecular weight markers (Roche). Electrophoresis pulse times for *Xba*I-digested DNA were 10 to 60 seconds for 24 hours, followed by a second ramp from 5 to 20 seconds for 5 hours. Both ramps were performed at 5.4 V/cm and 12º C. For *Spe*I, pulse times were 25 to 45 seconds for 20 hours at 6 V/cm and 12°C. Macrorestriction fragments were visually compared and interpreted according to the criteria of Tenover et al. (14).

A genetic similarity dendogram was designed and calculated by the Dice correlation coefficient (15) and represented by UPGMA with Molecular

Analyst Software (BioRad) and a tolerance position of 1%. Only well-resolved bands corresponding to fragments exceeding 97.0 kbp were included in the computer analysis.

### Patient Data

Chart records from *S. maltophilia*-positive CF patients were reviewed. Patients were classified according to age, sex, and severity of lung disease. Correlation between colonization or infection with *S. maltophilia* and pulmonary function was studied. Pulmonary function was tested in accordance with American Thoracic Society Guidelines (16). Forced expiratory volume ($FEV_1$) (% predicted) value was expressed as the percentage predicted according to Knudson norms for adjusting data for age, height, and sex (17). Trends in $FEV_1$ were estimated by comparing values at the time of the first recovery of *S. maltophilia* with those obtained within a year from the last isolation. *P. aeruginosa* and other pathogens commonly encountered in CF were also recorded as outcome criteria for evaluating the progression of pulmonary disease.

### Statistic Analysis

Statistical significance for comparison proportions was calculated by Chi square or Fisher's exact test with Epi-Info 6.04a. Quantitative values were compared by Student's *t* test; $p<0.05$ was considered statistically significant.

### Results

From 1991 to 1998, at least one respiratory culture positive for *S. maltophilia* was observed in 25 of 104 patients. Thus, the overall incidence of *S. maltophilia*-infected or -colonized patients was 24%; yearly incidence was 2.9% to 14.0% (Figure 1). Fourteen (56%) of these 25 patients had a single episode of *S. maltophilia* (SM-SE group), and 11 patients (44%) had repeated episodes (SM-RE group). No differences in sampling frequency (number of sputum samples studied per year) or length of follow-up were found between the two groups.

Eighty-eight *S. maltophilia* isolates were recovered from these 25 patients. Seventy-six isolates, 14 from the SM-SE group and 62 from the SM-RE group, were available for further study. PFGE results indicated an overall clonal incidence of 47.1%, reflecting new strains with different PFGE profiles that had been acquired by the SM-RE group (Figure 1). The highest yearly clonal incidences were detected in 1991 and 1996.

In the SM-SE group, the median age at acquisition of *S. maltophilia* was 13.4 years (range <1 to 27 years). Nearly 43% of patients (6 of 14) acquired *S. maltophilia* at 6 to 10 years of age (Figure 2). In SM-RE patients, the median age at first *S. maltophilia* isolation was 16.7 years (range 3 to 32 years). In this group, 45% (5 of 11) acquired *S. maltophilia* at 11 to 20 years of age. PFGE analysis of all *S. maltophilia* strains

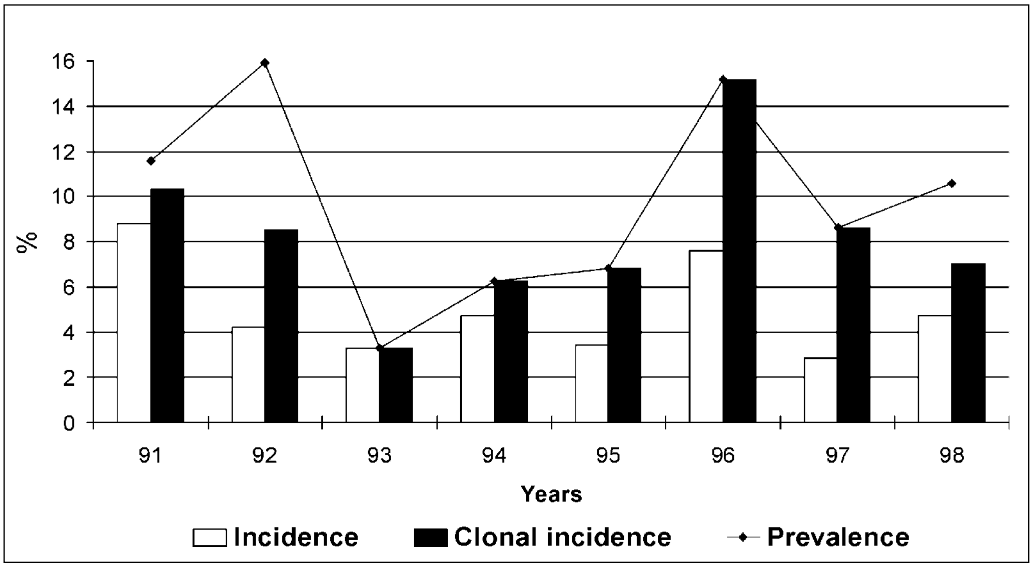

Figure 1. Yearly incidence, yearly clonal incidence, and yearly prevalence of *Stenotrophomonas maltophilia* acquisition in 104 cystic fibrosis patients, 1991–1998.

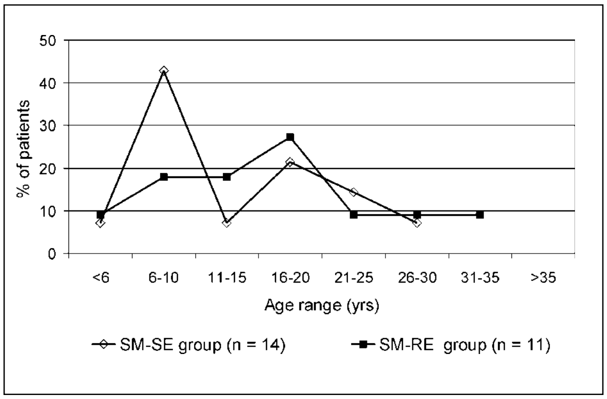

Figure 2. Distribution of age of acquisition of *Stenotrophomonas maltophilia* in 25 cystic fibrosis patients with a single episode (SM-SE group) or with repeated episodes (SM-RE group).

indicated that nine new acquisitions occurred in 11- to 15-year-old patients. Because of the small sample size, differences in age of acquisition between the groups could not be demonstrated with statistical significance.

### Ribotyping

To select the suitable enzyme(s) for *S. maltophilia* ribotyping, *Bam*H1, *Bsu*15I, *Eco*RI, and *Hin*dIII endonucleases were used in five different strains isolated from the same patient, resulting in 4, 4, 2, and 4 different ribotypes, respectively. The number of copies of the ribosomal rRNA operon in *S. maltophilia* was 2 to 5 per isolate for *Bam*HI, 2 to 4 *Bsu*15I for *Hin*dIII, and 4 to 5 for *Eco*RI, with hybridization band sizes of 3 Kbp to 20 kbp. Great heterogeneity in ribotypes, 21 with *Hin*dIII and 20 with *Bam*HI, was found among the 76 *S. maltophilia* isolates, with a Simpson index (15) of 0.8992 and 0.9158, respectively. The genetic similarity was 29% to 100% for *Hin*dIII and 38% to 100% for *Bam*HI.

### PFGE Analysis

Forty-seven well-defined profiles of genomic DNA under *Xba*I digestion were obtained from the 76 *S. maltophilia* isolates. According to Tenover criteria (14), 41 types and 6 subtypes were considered. These 6 subtypes were associated with 3 of the 41 main subtypes. Fragment size was <48 kbp to >1,000 kbp. Discrimination based on Simpson´s index peaked at 0.97. Genetic heterogeneity is illustrated by the dendogram of the 47 *Xba*I-PFGE profiles (Figure 3). Repeated isolates displaying an identical PFGE profile from

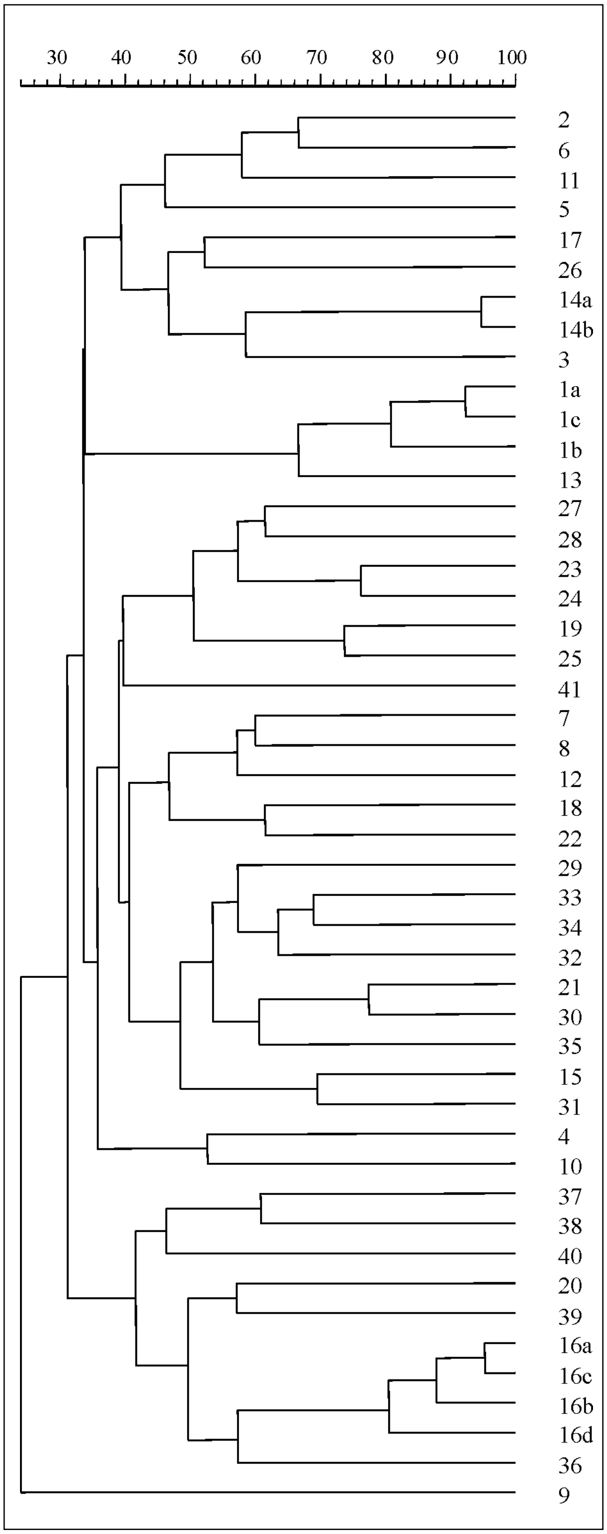

Figure 3. Percentages of genetic similarity between 47 *Xba*I-PFGE clonal types from 76 *Stenotrophomonas maltophilia* strains isolated in 25 cystic fibrosis patients of the same unit, 1991–1998.

the same patient or resulting from presumed patient-to-patient transmission were excluded from the dendogram. Forty-one types displayed similarity coefficients from 25% to 75%; each was coded with a number. Each subtype was coded with a letter (similarity ≥80%). Strains sharing the same *Xba*I digestion pattern could not be further distinguished by *Spe*I. *Xba*I was more efficient than *Spe*I in distinguishing between subtypes or closely related strains; 14a and 14b subtypes showed an indistinguishable PFGE pattern with *Spe*I. This was also the case with 16a and 16c subtypes.

### Persistence and Variability of *S. maltophilia* Strains

The SM-SE group of 14 patients had 14 different PFGE types. One of these PFGE patterns (pattern *1a*) was also seen in two of the SM-RE patients (patients 1 and 3). During the study period, each of the 11 patients in the SM-RE group had one to five strains with different PFGE profiles. Strains from five patients (1, 3, 8, 10, and 11) completely met the criteria for persistence (Figure 4). The strains were recovered from these patients during periods of persistence of 29, 86, 6, 9, and 8 months, respectively. A turnover of this predominant strain with a different strain occurred in four of these patients. In patient 4, two strains with 4 and 32 months of persistence were isolated during two different periods. All these patients were considered persistently colonized with identical *S. maltophilia* isolates (Figure 4). Variable colonization, defined as the isolation of *S. maltophilia* strains with different PFGE

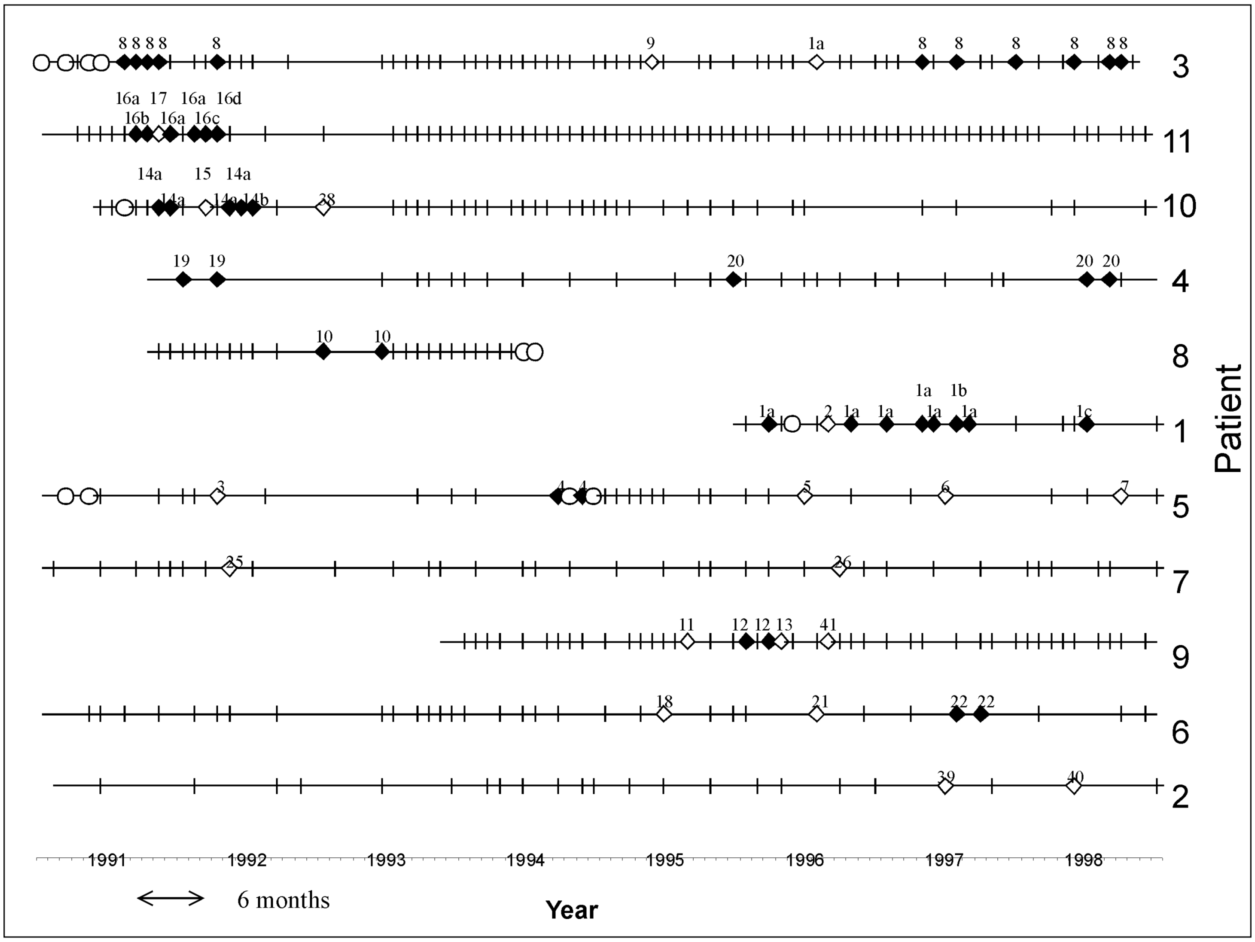

Figure 4. Persistence and variability of *Stenotrophomonas maltophilia* strains in 11 patients with repeated episodes of infection or colonization with this organism. *S. maltophilia* recovered is indicated by a ♦ (identical isolates from the same patient), ◊ (different isolate from a single patient) or O (no isolate available). Distinct *S. maltophilia* isolates are identified by numbers, and a letter was added when a closely related strain was observed. + = time at which sputum specimens were obtained.

profiles, was identified in five patients (patients 2, 5, 6, 7, and 9).

### Suspected Cross-Transmission

In 1996, three patients, two in the SM-RE group (patients 1 and 3) and another (patient 12) in the SM-SE group, shared *S. maltophilia* isolates with indistinguishable ribotype and PFGE type under all restriction enzymes tested (profile *1a*). Patient 1 was persistently colonized with this strain for 2 years, and patient 3 was transiently colonized (Figure 4).

### Bacterial Counts and Clinical Findings

SM-SE patients had higher *S. maltophilia* bacterial counts when either the first *S. maltophilia* isolate (geometric mean, 4.3 x$10^5$ cfu/mL) or all isolates (2.9 x$10^8$ cfu/mL) were taken into account ($p<0.05$), compared with patients with a single episode (8.4 x$10^4$ cfu/mL). A similar rate of *P. aeruginosa* recovered from the respiratory tract during the study period was noted in both groups (Table). In contrast, *Aspergillus* spp. was detected more frequently in the SM-RE group of patients. No statistical differences were found when co-colonization was evaluated. However, *S. maltophilia* co-colonization with *Aspergillus* spp. in the SM-RE group had a risk ratio of 3.8 compared with the SM-SE group.

Demographic and selected medical characteristics and results of respiratory tract cultures were analyzed for *S. maltophilia*-infected or -colonized patients (Table). Before *S. maltophilia* colonization, slightly lower pulmonary function levels ($FEV_1$, % predicted) were observed in patients with a single *S. maltophilia* episode than in patients with repeated episodes (Table).

Table. Demographic characteristics and co-colonization status of cystic fibrosis patients with *Stenotrophomonas maltophilia* infection or colonization

| Characteristics | SM-SE[a] (14 patients) | SM-RE[b] (11 patients) |
|---|---|---|
| Gender | | |
| Male | 8 | 5 |
| Female | 6 | 6 |
| Patient genotyped | 11 | 9 |
| Homozygous ΔF508 | 8 | 3 |
| Heterozygous ΔF508 | 3 | 5 |
| Other | 0 | 1 |
| Mean age at first *S. maltophilia* isolation (SD, years) | 13.4 (7.3) | 16.7 (7.4) |
| $FEV_1$[c] measured | 11 | 10 |
| $FEV_1$ (% predicted) before *S. maltophilia* recovery (Mean [SD]) | 68.7 (29.6) | 74.2 (28.3) |
| ≥100 | 2 | 1 |
| 70–99 | 3 | 5 |
| 40–69 | 4 | 3 |
| <40 | 2 | 1 |
| $FEV_1$ (% predicted) after *S. maltophilia* recovery (Mean [SD]) | 63.8 (20.7) | 62.9 (24.2) |
| ABPA[c] condition | 2 | 1 |
| Death (%) | 4 (28.5) | 2 (18.2) |
| *S. maltophilia* bacterial counts (geometric mean, cfu/mL) | 8.4 x $10^4$ [d] | 2.9 x $10^8$ [d] |
| *Pseudomonas aeruginosa* detected (%) | 12 (85.7) | 9 (81.8) |
| Aspergillus detected (%) | 7 (50.0) | 7 (63.6) |
| *S. maltophilia* co-colonization with:[e] | | |
| Only *S. maltophilia* detected (%) | 1 (7.1) | 1 (9.1) |
| *P. aeruginosa* (%) | 8 (57.1) | 3 (27.2) |
| *Staphylococcus aureus* (%) | 6 (42.8) | 3 (27.2) |
| *Burkholderia cepacia* (%) | 1 (7.1) | 1 (9.1) |
| *Aspergillus* spp. (%) | 1 (7.1) | 3 (27.2) |
| Candida (%) | 3 (21.4) | 2 (18.2) |

[a]SM-SE = CF patients with a single episode of *S. maltophilia* colonization.
[b]SM-RE: CF patients with repeated episodes of *S. maltophilia* colonization.
[c]FEV = Forced expiratory volume.
[d]ABPA: allergic bronchopulmonary aspergillosis.
[e]$p<0.05$ comparing both groups.
[f]Patients in the SM-RE group colonized with organisms in addition to *S. maltophilia*. When different co-colonizations occurred in the same patient, we recorded only the cocolonization that was at least twice as frequent.

This value decreased in SM-RE patients from 74.2 ± 28.3 (mean value ± SD) (first isolation of *S. maltophilia*) to 62.9 ± 24.2 (last isolation of *S. maltophilia*), which could indicate a decreasing trend in $FEV_1$ after the first episode. However, this difference was not statistically significant. On the other hand, SM-SE patients had a higher death rate (28.5%) than the SM-RE group, but death rates in both groups were higher than those observed in *S. maltophilia*-negative patients (12.6%).

### Conclusions

*S. maltophilia*, an essentially environmental organism, is the fourth organism in prevalence in bronchial secretions of CF patients, after *P. aeruginosa, Staphylococcus aureus,* and *Haemophilus influenzae* (5,18). Since it was first reported in CF patients in 1979 (19), this organism has been investigated for its role in the progression of CF pulmonary disease (5), and consensus documents have emphasized the importance of clinical microbiology laboratories in detecting its presence in CF respiratory secretions (20). Despite some virulence factors shared with *P. aeruginosa*, its potential for pathogenicity remains uncertain (21). We have reported a high incidence of *S. maltophilia*-colonized CF patients (30.7%) over a 5-year period (3), but, as in other studies (2,6,22), we did not address (through epidemiologic typing studies) whether this high rate was a consequence of patient-to-patient transmission or whether bacterial colonization was sporadically or chronically established.

The 1997 Cystic Fibrosis Foundation Patient Registry from the United States (18), which included 17,996 CF patients in a cross-sectional study analyzing one respiratory sample per patient per year, showed a percentage of positive cultures for *S. maltophilia* of 5.1%, a value slightly higher than in 1996 (3.9%) and 1995 (3.4%). In our study, the overall incidence, 24%, is higher than that observed in other studies (10.6% to 16.6%) with a similar length of follow-up (2,22), but slightly lower than in studies with a longer follow-up period (27.3%) (6). Consistent with other results, our data showed no clear trend towards increasing or decreasing over the study period (Figure 1).

The main purpose of our study was to apply molecular typing, both with ribotyping and PFGE, to *S. maltophilia* isolates recovered from patients seen in our CF Unit. Among 76 isolates, 47 PFGE profiles were identified, and these results were used to calculate the incidence of episodes of *S. maltophilia* colonization or infection in our series. Without typing, the overall incidence was 24% for the entire study period; by PFGE the incidence was 47.1%. This result clearly indicates that SM-RE patients had new episodes with different *S. maltophilia* strains. Molecular typing also differentiated patients who were chronically infected or colonized with the same strain (persistence) from those with repeated episodes with different *S. maltophilia* strains (variability).

PFGE has been recommended for epidemiologic studies of *S. maltophilia* isolates (13,23-25). The technique has been shown to be more discriminatory than enterobacterial repetitive intergenic consensus polymerase chain reaction and other molecular techniques for differentiation within this species (13). In our study, restriction endonuclease *Xba*I provides discriminatory patterns, with a high discrimination value on Simpson´s index (0.97), enabling easy interpretation of banding profiles. This enzyme has been used to study the stability of *S. maltophilia* from a CF patient over a 15-month period (26), the relationship between CF and environmental *S. maltophilia* isolates (13), and the epidemiology of *S. maltophilia* isolates from a hematology department (27). Other studies have been based on *Dra*I (25,28,29) and *Spe*I (23,27,30). In our study, *Xba*I was more efficient than *Spe*I in distinguishing between subtypes or closely related strains.

We observed only one positive culture of *S. maltophilia* over the study period in 14 patients, in accordance with the results of Demko et al. (6), who showed that 50% of CF patients had only one positive culture of *S. maltophilia* over a 13-year period. In contrast, 11 patients (44%) from our CF unit had repeated episodes of *S. maltophilia* colonization or infection. Typing studies, however, demonstrated different strains in five patients and, with the exception of patient 8, a persistent strain was characterized in the remaining six patients, but with a turnover with distinct strains (Figure 4). Because of sampling bias, some of these patients may also have had persistent colonization. Of 11 patients with repeated SM-RE isolates, 6 had evidence of persistent colonization (Figure 5). More frequent sampling could have increased this proportion.

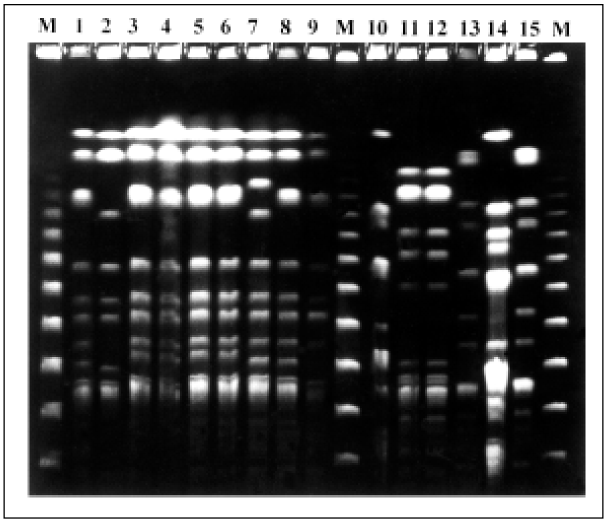

Figure 5. Pulsed-field gel electrophoresis pattern of *Xba*I-digested genomic DNA of *Stenotrophomonas maltophilia* isolates from two SM-RE patients. Lanes 1-10 from patient 1 (persistence group): pattern 1a (lanes 1,3-6,8), pattern 1b (ln 7), pattern 1c (lane 9) and pattern 2 (lane 2); lanes 10-15 from patient 5 (variability group): pattern 3 (lane 10), pattern 4 (lane 11,12), pattern 5 (lane 13), pattern 6 (lane 14), pattern 7 (lane 15). Lanes M, bacteriophage lambda standard marker.

Cross-transmission was suspected in three patients who shared isolates with an identical PFGE profile. No overlapping hospitalizations, clinical visits, or other epidemiologic relationship were demonstrated in these patients. Recently, Alfieri et al. (30) reported cross-transmission of *S. maltophilia* in non-CF patients during two consecutive nosocomial outbreaks in an intensive care unit, but an environmental ventilator isolate was temporally associated with infection.

Heterogeneity is also illustrated among *S. maltophilia* isolates recovered from the same patient. SM-RE patients 2, 5, 6, 7, and 9 were colonized at different times by different clones with PFGE similarity genetic coefficients of 24% to 61%. Among *S. maltophilia* isolates recovered from different patients, the genetic coefficient range was even wider (25% to 75%). This heterogeneity could result from acquisition from different environmental sources, probably outside the nosocomial setting. In fact, a high diversity of *S. maltophilia* isolates has also been confirmed in the environment (13,25). The precise mode of acquisition of *S. maltophilia* in CF patients has not been determined, but different studies strongly suggest that faucets, ventilators, sink drains, and other devices frequently in contact with water could be common sites of contamination (13,25,28,30,31).

In most cases, chronic colonization with *P. aeruginosa* occurs with a single strain, which undergoes phenotypic variation over time (32). This changing adaptive response is probably driven by stressful conditions of the lung environment for bacterial organisms and results from the selection of hypermutable genetic variants (33). In the case of *S. maltophilia*, the isolation of the same clonal type after years of apparent absence suggests a long low-grade persistence that could not be detected by microbiologic culture. In patient 3, the same strain was isolated 11 times over a 7-year period without change in its PFGE profile. The differing subtypes in patients 1, 10, and 11 may be accounted for by genetic events during chronic colonization (Figure 4).

The 1997 Cystic Fibrosis Patient Registry Annual Report (18) showed that *S. maltophilia* respiratory colonization was 3.1% to 8.6% in patients 2 to 5 and >45 years of age, respectively, with a clear increase in patients >35 years of age. We analyzed the age at first acquisition of an *S. maltophilia* isolate, including all 25 patients with at least one positive culture for this organism during the study period. When available, a retrospective review of cultures obtained before 1991 was also taken into account. Colonization rates were 4% to 24% in the 31-35 and 16-20 age groups, respectively. The peak age of acquisition was 16-20 years, as reported by Demko et al. (6), but the two groups of *S. maltophilia*-colonized patients, SM-SE and SM–RE, differed in age of acquisition. In SM-SE patients, peak age of acquisition was 6 to 10 years (42.8%); in the SM–RE group it was 16 to 20 years (27.2%). These results suggest that *S. maltophilia* colonization in younger CF patients could be an isolated event, whereas chronic colonization with this organism occurs more frequently when acquired in 16- to 20-year-old patients.

Higher significant ($p<0.05$) differences in *S. maltophilia* bacterial counts were obtained in patients persistently colonized with this organism compared with those with single episodes, suggesting that the colonizing ability of a given strain may be a marker for future persistence. In addition, the former group had a decline in pulmonary function as indicated by $FEV_1$ (% predicted) values closest to the first and last *S. maltophilia* isolations. Reduction in pulmonary function could also reflect increased age or the effect of other pathogens. In fact, a higher rate of *Aspergillus* spp. isolation was detected in CF patients chronically colonized with *S. maltophilia*. However, a higher death rate was observed in patients with a single episode of *S. maltophilia* (28.5%) than in patients with repeated episodes (18.2%), but both these values were higher than those obtained in *S. maltophilia*-negative patients (12.6%). Demko et al. recently reported a lower death rate in patients with long-term chronically *S. maltophilia*-positive cultures (7.7%) than in those with transient or acute positive cultures (21.1%)(6). Moreover, the combined death rate in *S. maltophilia*-positive patients (19.0%) was slightly higher than in *S. maltophilia*-negative patients (16.5%). In contrast, Goss et al. (34) demonstrated in a cohort study that *S. maltophilia* acquisition did not decrease survival in patients with CF, but patients with this organism had significantly lower $FEV_1$ (% predicted) values. These data suggest that isolation and persistence of *S. maltophilia* could contribute to a progression of clinical deterioration, particularly in patients with lower pulmonary function. Increased *S. maltophilia* colonization may be observed in the future as a result of improvements in life expectancy.

This work was supported by research grant 2114/98 from the Consejería de Educación y Cultura of the Comunidad de Madrid, Spain.

Dr. Valdezate is a fellow in the Clinical Microbiology Department at the Ramón y Cajal Hospital and at the Centro Nacional de Microbiología (Instituto Carlos III) in Madrid, Spain. Her research interests focus on epidemiology and resistance of *Stenotrophomonas maltophilia*, mainly from cystic fibrosis patients.

### References

1. Gladman G, Connor PJ, Williams RF, David TJ. Controlled study of *Pseudomonas maltophilia* in cystic fibrosis. Arch Dis Child 1993;67:192-5.
2. Karpati F, Malmborg AS, Alfredsson H, Hjelte L, Strandvik B. Bacterial colonization with *Xanthomonas maltophilia*: a retrospective study in a cystic fibrosis patient population. Infection 1994;22:258-63.
3. Ballestero S, Virseda I, Escobar H, Lucrecia L, Baquero F. *Stenotrophomonas maltophilia* in cystic fibrosis patients. Eur J Clin Microbiol Infect Dis 1995;14:728-79.
4. Burns JL, Emerson J, Stapp JR, Yim DL, Krzewinski J, Louden L, et al. Microbiology of sputum from patients at cystic fibrosis centers in the United States. Clin Infect Dis 1998;27:158-63.
5. Denton M. *Stenotrophomonas maltophila*: an emerging problem in cystic fibrosis patients. Rev Med Microbiol 1997;8:15-9.
6. Demko CA, Stern RC, Doershuk CF. *Stenotrophomonas maltophilia* in cystic fibrosis: incidence and prevalence. Pediatr Pulmonol 1998;25:304-8.
7. Villarino ME, Stevens LE, Schable B, Mayers G, Miller JM, Burke JP, et al. Risk factors for epidemic *Xantomonas maltophilia* infection/colonization in intensive care unit patients. Infect Control Hosp Epidemiol 1992;13:201-6.
8. Van Couwenberghe CJ, Farver TB, Cohen SH. Risk factors associated with isolation of *Stenotrophomonas (Xantomonas) maltophilia* in clinical specimens. Infect Control Hosp Epidemiol 1997;18:316-21.
9. Wong K, Roberts MC, Owens L, Fife M, Smith AL. Selective media for the quantification of bacteria in cystic fibrosis sputum. J Med Microbiol 1984;17:113-9.
10. Ausubel FM, Brent R, Kingston RE, Moore DD, Seidman JG, Smith JA, et al. Current protocols in molecular biology. New York: Greene Publishing Associates and Wiley Interscience; 1991( Suppl. 13):241-5.
11. Southern EM. Detection of specific sequences among DNA fragments separated by gel electrophoresis. J Mol Biol 1975; 98:503-17.
12. Stull TL, LiPuma JJ, Edlind TD. A broad-spectrum probe for molecular epidemiology of bacteria: ribosomal RNA. J Infect Dis 1988;157:280-6.
13. Denton M, Todd NJ, Kerr KG, Hawkey PM, Littlewood JM. Molecular epidemiology of *Stenotrophomonas maltophilia* isolated from clinical specimens from patients with cystic fibrosis and associated environmental samples. J Clin Microbiol 1998;36:1953-8.
14. Tenover FC, Arbeit RD, Goering RV, Mickelsen PA, Murray BE, Persing DH, et al. Interpreting chromosomal DNA restriction patterns produced by pulse-field gel electrophoresis: criteria for bacterial strain typing. J Clin Microbiol 1995;33:2233-9.
15. Hunter PR, Gaston MA. Numerical index of the discriminatory ability of typing systems: an application of Simpson‘s index of diversity. J Clin Microbiol 1988;26:2465-6.
16. American Thoracic Society. Standardization of spirometry: 1987 update. Am Rev Respir Dis 1987;136:1285-98.
17. Knudson RJ, Lebowitz MD, Holdberg CJ, Burrows B. Changes in the normal maximal expiratory flow-volume curve with growth and aging. Am Rev Respir Dis 1983;127:725-34.
18. National CF Patient Registry 1997 Annual Report. Bethesda, MD: Cystic Fibrosis Foundation; 1998.
19. Blessing J, Walker J, Maybury B, Yeager AS, Lewiston N. *Pseudomonas cepacia* and *Pseudomonas maltophilia* in cystic fibrosis patients [abstract]. Am Rev Respir Dis 1979;119:262.
20. Gilligan P. Report on the consensus document for microbiology and infectious diseases in cystic fibrosis. Clin Microbiol Newsletter 1996;18:83-7.
21. Denton M, Kerr KG. Microbiology and clinical aspects of infection associated with *Stenotrophomonas maltophilia*. Clin Microbiol Rev 1998;11:57-80.
22. Gladman G, Connor PJ, Williams RF, David TJ. Controlled study of *Pseudomonas cepacia* and *Pseudomonas maltophilia* in cystic fibrosis. Arch Dis Child 1992;67:192-5.

23. Laing FPY, Ramotar K, Read RR, Alfieri N, Kureishi A, Henderson EA, et al. Molecular epidemiology of *Xanthomonas maltophilia* colonization and infection in the hospital environment. J Clin Microbiol 1995;33:513-8.
24. Marty N. Epidemiological typing of *Stenotrophomonas maltophilia*. J Hosp Infect 1997;36:261-6.
25. Berg G, Roskot N, Smalla K. Genotypic and phenotypic relationships between clinical and environmental isolates of *Stenotrophomonas maltophilia*. J Clin Microbiol 1999;37:3594-600.
26. Wüst J, Frei R, Gunthard H, Altwegg M. Analysis of restriction fragment length polymorphism and ribotyping of multirresistant *Stenotrophomonas maltophilia* isolated from persisting lung infection in a cystic fibrosis patient. Scand J Infect Dis 1995;27:499-502.
27. Fabe C, Rodríguez P, Cony-Makhoul P, Parneix P, Bebear C, Maugein J. Typage moleculaire par electrophorese en champ pulse de souches de *Stenotrophomonas maltophilia* isolees dans un service d'hematologie. Pathol Biol Paris 1996; 44:435-41.
28. Talon D, Bailly P, Leprat R, Godard C, Deconnink E, Cahn J-Y, et al. Typing of hospital strains of *Xanthomonas maltophilia* by pulse-field gel electrophoresis. J Hosp Infect 1994;27:209-17.
29. Yao JD, Conly JM, Krajden M. Molecular typing of *Stenotrophomonas (Xanthomonas) maltophilia* by DNA macrorestriction analysis and random amplified polymorphic DNA analysis. J Clin Microbiol 1995;33:2195-8.
30. Alfieri N, Ramotar K, Armstrong P, Spornitz ME, Ross G, Winnick J, et al. Two consecutive outbreaks of *Stenotrophomonas maltophilia (Xanthomonas maltophilia)* in an intensive-care unit defined by restriction fragment-length polymorphism typing. Infect Control Hosp Epidemiol 1999;20:553-6.
31. Weber DJ, Rutala WA, Blanchet CN, Jordan M, Gergen MF. Faucet aerators: A source of patient colonization with *Stenotrophomonas maltophilia*. Am J Infect Control 1999;27: 59-63.
32. Govan JRW, Nelson JW. Microbiology of lung infection in cystic fibrosis. Br Med Bull 1992;48:912-30.
33. Oliver A, Cantón R, Campo P. Baquero F, Blázquez J. High frequency of hypermutable *Pseudomonas aeruginosa* in cystic fibrosis. Science 2000;288:1251-3.
34. Goss CH, Aitken ML, Johnson WC, Campbell PW, Rubenfeld GD. Acquiring *Stenotrophomonas maltophilia* does not decrease survival in patients with cystic fibrosis. Pediatr Pulmunol 1999(Suppl 19):334-335 (abstract)
